# What’s the Link Between Theory of Mind and Other Cognitive Abilities – A Co-twin Control Design of Neurodevelopmental Disorders

**DOI:** 10.3389/fpsyg.2021.575100

**Published:** 2021-06-08

**Authors:** Johan Isaksson, Janina Neufeld, Sven Bölte

**Affiliations:** ^1^Department of Neuroscience, Child and Adolescent Psychiatry Unit, Uppsala University, Uppsala, Sweden; ^2^Center of Neurodevelopmental Disorders (KIND), Centre for Psychiatry Research, Department of Women’s and Children’s Health, Karolinska Institutet and Stockholm Health Care Services, Region Stockholm, Stockholm, Sweden; ^3^Curtin Autism Research Group, Curtin School of Allied Health, Curtin University, Perth, WA, Australia; ^4^Child and Adolescent Psychiatry, Stockholm Health Care Services, Region Stockholm, Stockholm, Sweden

**Keywords:** social cognition, theory of mind, central coherence, executive function, intelligence, twin study, autism, ADHD

## Abstract

Theory of mind (ToM), or the ability to attribute mental states to oneself and others, is a core element of social cognition (SC). Even though its importance for social functioning in general, and neurodevelopmental disorders (NDDs), in particular, is well established, the links between ToM and other cognitive functions are not. Especially the familial underpinnings of such links remain unclear. Using a co-twin control design, we examined *N* = 311 twins (mean age *M* = 17.19 years, 47% females) diagnosed with autism spectrum disorder (ASD), attention-deficit/hyperactivity disorder (ADHD), other NDDs, or typically developing individuals. We used the Reading the Mind in the Eyes Test to operationalize ToM, the Fragmented Pictures Test for central coherence (CC), the Tower Test for executive functioning (EF), and the general ability index in the Wechsler Intelligence Scales for IQ. In the linear regressions, weak CC and a lower IQ were associated with a reduced ToM ability across pairs. Female sex and higher age were robustly associated with increased ToM ability, whereas EF was not associated with ToM. In the within-pair analyses, where unmeasured familial confounders are implicitly adjusted, the associations between ToM and other cognitive functions, were attenuated and the association with CC was non-significant. The result suggests that familial factors shared by the twins, such as genetic and shared environment, influence the association between CC, IQ, and ToM. Future studies need to include a larger sample of monozygotic twins, who are genetically identical, in order to draw more firm conclusions regarding the influence of familial factors, and to differentiate between shared environmental and genetic effects on the associations between cognitive functions.

## Introduction

Social cognition (SC) is presumed to form the basis of human social interaction and communication (Happé et al., 2017). SC encompasses a wide range of interrelated processes and skills, such as social motivation, social awareness, emotion recognition, social attention, and social learning (Happé et al., 2017). Still Theory of Mind (ToM) or the ability to mentalize around one’s own and others thoughts, emotions and beliefs, might constitute the core element of SC, and is commonly also referred to as cognitive empathy (Grove et al., 2014; Happé et al., 2017). Accordingly, ToM has been associated with a wide range of social functioning outcomes, including peer-popularity (Slaughter et al., 2015), social competence (Razza, 2009), and being a bully or a bully-victim (Shakoor et al., 2012). Alterations in ToM have foremost been observed among individuals with neurodevelopmental disorders (NDDs), particularly autism spectrum disorder (ASD) (Morgan et al., 2003; Callenmark et al., 2014; Baron-Cohen et al., 2015; Bölte et al., 2015; Atherton and Cross, 2018; Isaksson et al., 2019b), and to a lesser degree in attention-deficit/hyperactivity disorder (ADHD) (Baribeau et al., 2015; Mary et al., 2016) and communication and language disorders (Smit et al., 2019).

Even though the importance of SC and ToM for social functioning and NDDs is well established, its putative link to other cognitive functions, and especially the aetiological nature of their association, remains to be established. Besides being associated with IQ (Coyle et al., 2018), ToM has shown to be related to executive functioning (EF) and central coherence (CC) in general and NDD populations (Jarrold et al., 2000; Pellicano, 2010; Devine and Hughes, 2014; Pineda-Alhucema et al., 2018; Wade et al., 2018). EF refers to higher order cognitive processes involved in the control of thought and action, including planning, working memory, inhibitory control, cognitive flexibility, and set-shifting (Diamond, 2013). CC comprises the processing of information in a broader context and top-down style, whereas weak CC (WCC) results in a more detail-focused approach with a preference of local over global information (Happé and Frith, 2006). The interrelations between these cognitive functions have been increasingly studied, especially links between ToM and EF.

Theory of mind, EF, WCC, and (low) IQ are thought to re?ect underlying cognitive alterations within NDDs, although, to some degree, different cognitive functions have been associated with certain NDDs. For instance, it has been hypothesized that alterations in ToM augment the social and communication difficulties in ASD (Baron-Cohen, 2009; Mazza et al., 2017), that EF deficits contribute to core symptoms of ADHD (Alderson et al., 2007; Kasper et al., 2012) and rigid and repetitive behaviors in ASD (Demetriou et al., 2019). WCC might underlie uneven cognitive profiles in ASD such as autism related strengths and savant talents (Happé and Frith, 2006). Low IQ is the core definition of intellectual disorder (ID), and increases the symptom burden in a wide range of NDDs (Matson and Shoemaker, 2009). However, previous research on cognitive functions in NDDs has been somewhat limited to investigating single cognitive functions, e.g., ToM in ASD, not simultaneously including multiple cognitive functions or NDDs in the same models (Brunsdon et al., 2015), which may yield shortcomings given the considerable overlap between different types of NDDs (Licari et al., 2019).

ToM, EF, and CC mature throughout childhood and have been reported to intercorrelate during development in typically developing individuals (TD). EF, such as working memory, inhibition, and cognitive flexibility are correlated with ToM abilities, such as understanding false beliefs, and are usually basically established by school-age (Pineda-Alhucema et al., 2018). According to a developmental model, these systems are dependent and impairment in one function early on during development may have substantial knock-on effects on other cognitive functions (Pellicano, 2010). Consistently, ToM and EF have been found to share underlying neuroanatomical mechanisms (Wade et al., 2018). Although the degree of prediction and predictability of one cognitive function over the other is yet be determined (Pineda-Alhucema et al., 2018), there is preliminary support from longitudinal studies that both EF and CC are precursors of ToM performance in TD and individuals with ASD or ADHD (Pellicano, 2010; Devine and Hughes, 2014; Mary et al., 2016; Skorich et al., 2016; Wade et al., 2018). In line with the latter, it has also been hypothesized that ToM abilities rely on a general information processing system, including the integration of stimulus information into a coherent whole, i.e., CC, and mental flexibility, response inhibition, and working memory, i.e., EF in ASD (Pellicano, 2010). However, this notion has also been criticized and it has been argued that ToM and WCC should be seen as separate at a genetic and cognitive level, although co-occurring (Happé and Ronald, 2008; Brunsdon and Happé, 2014). Indeed, findings on the association between the different cognitive functions have ultimately rather been mixed in for example ASD populations (Pellicano et al., 2006). Thus, it remains to be elucidated if there are common underlying factors that drive alteration in these cognitive functions in NDDs.

An alternative approach to assess the putative link between ToM, EF, CC and IQ in NDDs is by examining the aetiological basis of their relations, i.e., the genetic and environmental influences on their associations, and whether these cognitive functions are influenced by similar familial factors. While previous research has mainly been conducted using a developmental approach and regressing ToM performance on CC, EF, and IQ in the general population- or clinical samples, there is a paucity of studied conducted in population-based twin samples enriched for NDDs. To the best of our knowledge, to date only one study has investigated the association between ToM, WCC, and EF in a twin-population consisting of children with ASD and TD co-twins and peers (Brunsdon et al., 2015). The authors found that children with ASD performed atypically on measures of ToM, EF, and WCC with 1/3 of ASD cases, as compared to 1/10 of the TD co-twins, having atypical performance in tasks across all three cognitive domains, a result indicating low levels of familial confounding. They did, however, compare affected twins to unaffected co-twins at a group level, rather than regressing within-pair differences in outcomes on within-pair differences in exposure, and did hence not apply a co-twin control approach.

Expanding on the study by Brundson et al. by including a broader range of NDDs and also applying a co-twin control design, our study enables more information on the aetiological basis by automatically controlling for factors shared between twins in a pair. In a previous study of our lab, we found a within-pair association between WCC in terms of reduced global visual processing and ASD diagnosis, suggesting that this relationship is not solely driven by familial factors (Neufeld et al., 2020). However, we did not investigate the association between WCC and ToM. Familial factors include genetic factors since dizygotic (DZ) twins share on average half of their genome and monozygotic (MZ) twins are genetically identical, as well as shared environmental factors (including parenting style, prenatal factors, and social environment during upbringing) in both types of twins. Familial factors may be adjusted for in a step-wise manner. First, by using a between-subject analysis, where all twins as treated as singletons, it is possible to first get an estimation of the associations investigated across the cohort. As a second step, applying within-pair analyses, adjustments are made 50% or 100% of the genome, respectively, and for all environmental exposures within the family that make the twins similar to each other. Lastly, by only including MZ twins in the within-pair analyses, all genetic factors are adjusted for as well. If ToM shares its aetiological basis with other cognitive abilities and is as such influenced by similar familial factors, the association between these abilities should be attenuated with each step. Any remaining association between ToM and other cognitive functions in the MZ co-twin design is therefore attributable to factors unique to an individual within the same family (i.e., non-shared environmental factors).

Thus, the aim of this study was to (i) investigate the putative link between ToM and other significant cognitive functions, namely CC, EF, and IQ within a sample of MZ and DZ twins enriched for twins concordant and discordant for NDDs, as well as TD control twins, and to (ii) explore if associations are driven by familial factors shared by twins (genetics and shared environment) or remain within (MZ) twin pair indicating non-shared environmental influence. The finding may provide a better understanding of common etiological pathways to altered crucial cognition functions in general and NDDs in particular.

## Materials and Methods

### Participants

The Roots of Autism and ADHD Twin Study Sweden (RATSS) (Bölte et al., 2014) is an ongoing study that includes twin pairs from the population-based Child and Adolescent Twin Study in Sweden (Anckarsäter et al., 2011) and the Young Adult Twins in Sweden Study (YATSS), where one or both twins have been screened positively for ASD or ADHD, as well as TD controls. Twins that are included in RATSS are comprehensively clinically phenotyped during a 2½ day visit at a clinical research unit. Zygosity is determined on a panel of 48 single nucleotide polymorphisms (Hannelius et al., 2007). In a few cases (22 pairs), where DNA results had not yet been analyzed, a 4-item zygosity questionnaire was used, and in 10 cases the zygosity was pending. From the total sample, 52 individuals were excluded due at least one of the twin missing data, 48 were excluded due to at least one twin having an intellectual disability or borderline intellectual functioning (IQ ≤ 75) and nine for having different sex. In the current study, *N* = 311 [170 MZ, 131 DZ (one triplets included), and 10 with pending zygosity] were included (46.9% females; mean age = 17.19 years, SD = 6.43, range: 8–36 years). In total, 125 had a NDD and of these 45 had two or more NDDs; 60 had a diagnosis of ASD (39 males, 21 females), 73 had a diagnosis of ADHD (50 males, 23 females), 51 other NDDs (e.g., communication disorders, specific learning disorders or motor disorders; 36 males, 15 females). Sample characteristics are summarized in Table 1. The study was approved by the Regional Swedish Ethical Review Board and Informed consent was obtained from all participants after the nature of the procedure had been fully explained.

**TABLE 1:** Sample characteristics and included factors.

	Pairs concordant	Typically
	or discordant for	developing
	NDDs *N* = 177	pairs *N* = 134
Sex (females)	35.0%	62.7%
Age (M,SD)	15.19 (5.68)	19.84 (6.41)
Parents civil status (Married)	58.8%	58.6%
Mother’s level of education		
Elementary school	5.7%	8.3%
Secondary school	56.0%	44.7%
University	38.3%	47.0%
Father’s level of education		
Elementary school	10.4%	13.1%
Secondary school	60.7%	52.3%
University	28.9%	34.6%
Work/study		
Mother	89.7%	91.9%
Father	93.9%	86.8%
Zygosity (Monozygotic)	42.9%	70.1%
ASD	33.9%	0%
ADHD	41.2%	0%
Other NDDs	28.8%	0%
Theory of Mind (M,SD)^a^	69.74 (12.95)	75.40 (11.85)
Central Coherence (M,SD)^b^	70.93 (6.60)	66.46 (6.45)
Executive Function^c^ (M,SD)	10.62 (2.52)	10.94 (2.18)
IQ/General cognitive abilities (M,SD)^d^	101.51 (13.04)	103.84 (12.73)

*^*a*^Measured with Reading the Mind in the Eyes Test, % correct answers. ^*b*^Measured with the Fragmented Pictures Test, no. of images for recognition. ^*c*^Measured with the Tower Test, scales scores. ^*d*^Measured with Wechsler Intelligence Scales for Children or Adults-IV. ADHD, attention-deficit/hyperactivity disorder; ASD, autism spectrum disorder; NDDs, neurodevelopmental disorders (other NDDs include e.g., communication disorders, specific learning disorders or motor disorders).*

### Diagnostic and Behavioral Assessments

A DSM-5 consensus diagnosis of any of the included NDDs were determined by a group of clinicians using a multitude of collected data, including medical history, diagnostic interviews and by first choice standardized diagnostic tools [for more detail see Bölte et al. (2014) and Isaksson et al. (2019a,b)]. These tools include structural interviews such as the Kiddie Schedule for Affective Disorders and Schizophrenia (Kaufman et al., 1997) or the Structured Clinical Interview for DSM-IV (SCID 1) depending on the participant’s age; autism-specific tools such as the Autism Diagnostic Interview—Revised (ADI-R; Rutter et al., 2003), the Autism Diagnostic Observation Schedule Second Edition (ADOS-2, modules 3 and 4, Lord et al., 2012), and the parent-report version of the Social Responsiveness Scale Second Edition (SRS-2, Constantino and Gruber, 2005); ADHD-related instruments are the Diagnostic Interview for ADHD in adults (Kooij and Francken, 2010) and the Conners Rating Scale 3rd Edition (Conners, 2008); and measure of adaptive functioning using the Adaptive Behavior Assessment System-2 (ABAS-2).

### Cognitive Functions

#### Theory of Mind

Theory of mind, as a construct within SC, was assessed with the Swedish version of the revised Reading the Mind in the Eyes Test (Söderstrand, 2006; Zander et al., 2011). Tasks aiming to measure alterations in SC have been criticized for not being able to assess more subtle alterations given their logical structure, encouraging a more deliberate reasoning (Callenmark et al., 2014). We selected the Reading the Mind in the Eyes Test as measure of ToM, which was developed to test ToM with sufficient sensitivity in both intellectually able individuals and in adults (Baron-Cohen et al., 1997, 2001). The test builds on the finding that the eye region is a hot spot for social communication information. The participants are presented with photographs of human eye regions portraying different emotions or mental states (e.g., playful/comforting/irritated/bored) and the participants are instructed to choose one word of four alternatives that most adequately matches the eye region’s expression. Reading the Mind in the Eyes Test is generally regarded as an advanced test of SC, as the participant is required to decode/attribute complex mental and emotional states, which promotes unconscious, rapid, and automatic processes (Baron-Cohen et al., 2001). The child version (14 ≤ years) contains 28 photos and the adult version (≥15 years) 36 photos. Expected alternatives are scored “1” and unexpected “0.” The number of correct answers was summed for each participant and a percentage of correct answers were calculated in order to enable merging the child and adult version. A higher score indicates a better ToM ability. The Reading the Mind in the Eyes Test has in previous literature shown diagnostic or discriminatory validity, foremost ASD vs. TD, but also ADHD vs. TD, for children and adults (Baron-Cohen et al., 2001, 2015; Losh et al., 2009; Sachse et al., 2014; Baribeau et al., 2015). The test has also shown good test–retest reliability (Hallerbäck et al., 2009), acceptable internal consistency and evidence for a single factor structure (Vellante et al., 2013).

#### Central Coherence

Central coherence was assessed with the Fragmented Pictures Test (Kessler et al., 1993). More specifically, the test assesses the ability to integrate elements of visual information into a meaningful whole with as little visual information as possible (global visual processing). The participants are presented fragmented drawings of 10 different objects. Each object is displayed in 10 sequential steps where each step reveals more visual information about the object (a more complete image). The participants are instructed to browse through the images keeping a steady pace, and to respond when they identified the object. The score was calculated as the sum of images needed across trials in order to identify the objects correctly, where a higher score indicates a need for more complete visual information and hence a WCC, i.e., a reduced ability for global processing. Results on the Fragmented Pictures Test have been shown to differ between individuals with autism compared to TD controls (Scheurich et al., 2010; Booth and Happé, 2018) where individuals with autism need more visual information in order to identify the object, indicating a WCC with a reduced drive to focus on the global gestalt of visual information (Happé and Frith, 2006). In this study, Cronbach’s alpha was 0.93, indicating a strong reliability in terms of internal consistency of the test.

#### Executive Function

Executive function was measured using the Tower Test, which is a cognitive test included in the Delis Kaplan Executive Function System (D-KEFS; Delis et al., 2001). The Tower Test is composed of a series of nine items, each one more difficult than the previous, and the test measures EF such as spatial planning, inhibition of impulsive and perseverative responding, establishment, and militainment of an instructional set. The participant is shown a picture of a tower, and instructed to move disks of various sizes across three pegs until the target tower is built, using as few numbers of moves as possible. Tower planning tasks are frequently used as a measure of EF, and in particular of deficient planning, involving the execution of cognitive and/or behavioral strategies required to attain a goal (Patros et al., 2019). In this study we used the total achievement score, i.e., number of moves, converted to scaled score with a higher score indicating fewer EF problems. Discriminant validity for the Tower Test regarding especially ADHD, but also ASD, has been reported in several studies (Craig et al., 2016; Patros et al., 2019), with individuals with NDDs requiring more moves to build the tower. The test has shown moderate to high internal consistency and moderate test–retest reliability (Delis et al., 2001).

#### IQ

The general ability index (GAI) of the Wechsler Intelligence Scales for Children or Adults-IV (WISC-IV/WAIS-IV) was used to assess IQ. The GAI is a composite score that is based on three Verbal Comprehension (i.e., Vocabulary, Comprehension, and Similarities) and three Perceptual Reasoning (i.e., Block Design, Matrix Reasoning, and Picture Concepts) subtests. The score does not include the Working Memory or Processing Speed subtests that are included in the Full Scale IQ. The GAI provides information about higher-order thinking abilities, as compared to the Working Memory or Processing Speed tests that provide information of cognitive processing proficiency. GAI has been shown to have very high reliability (Saklofske et al., 2010). A higher GAI score indicates a higher general IQ.

### Data Analyses

Associations between the study variables were first assessed using correlations (Spearman’s rho for continuous variables, the point-biserial correlation coefficient between a binary and continuous variable, and the phi correlation coefficient between binary variables). As sensitivity analyses, correlations between subtests of the IQ measures and cognitive abilities, including ToM, were explored.

In the main analysis, linear regressions in a generalized estimating equations (GEE) framework were used that fully account for twin/co-twin designs and allowing both categorical and continuous data (Neuhaus and McCulloch, 2006), using the drgee package (v.1.1.10) in R (v. 3.5.1). The main analyses in the GEE were conducted in several steps. First, we estimated associations between CC, EF, and IQ as independent variables, and ToM as an outcome across pairs (i.e., twins were treated as individuals but standard errors were adjusted for twin clustering), also adjusting for age and sex, in three separate models (i.e., for CC, EF, and IQ separately). Results are presented for the whole sample and split by NDD (concordant or discordant pairs) and TD pairs. Second, we included all cognitive functions (CC, EF, and IQ), NDD diagnoses (ASD, ADHD, and other NDDs), sex and age as independent variables within the same model. Third, we repeated the analyses at step 1 and 2 within the pairs in order to also adjust for unmeasured familial confounders. In this third step, each pair is considered a separate stratum where within-pair differences in outcomes are regressed on within-pair differences in exposure, while the models implicitly adjust for shared environmental factors and at least 50% of genetic factors. Finally, we re-calculated within-pair analyses in the MZ sub-cohort, in order to investigate the robustness of results when genetic confounding was completely adjusted, and any remaining association in the MZ subpopulation must therefore be influenced by non-shared environmental factors. Two tailed tests with *p* values < 0.05 were considered significant.

## Results

### Associations Between ToM and the Other Study Variables

Correlations between the study variables are presented in Table 2, showing that ToM ability was positively associated with EF and IQ, and negatively associated with CC. In addition, female sex and older age was positively associated with ToM, whereas ASD, ADHD and other NDDs were negatively associated with ToM. As a sensitivity analysis, exploring subtests of the IQ measures, the correlations with the cognitive abilities largely remained for the Verbal Comprehension and the Perceptual Reasoning subtests (Supplementary Table 1).

**TABLE 2:** Correlations between cognitive functions, sex, age and NDDs.

	ToM	CC	EF	IQ	Sex (male)	Age	ASD	ADHD
CC	−0.389***							
EF	0.198***	−0.129*						
IQ	0.312***	−0.198***	0.283***					
Sex (male)	−0.332***	0.271***	–0.082	–0.022				
Age	0.448***	−0.605***	0.141*	0.043	−0.330***			
ASD	−0.197***	0.229***	–0.084	–0.053	0.117*	−0.141*		
ADHD	−0.229***	0.247***	–0.072	–0.027	0.171**	−0.272***	0.287***	
Other NDDs	−0.151**	0.128*	−0.127*	–0.107	0.156**	−0.178**	0.202***	0.267***

Correlations between continuous variables were calculated using Spearman’s Correlation Coefficient, between a binary and continuous variable using Point-Biserial Correlation Coefficient, and between two binary variables the Phi Correlation Coefficient; ADHD, attention-deficit/hyperactivity disorder; ASD, autism spectrum disorder; CC, central coherence; EF, executive functions; GAI, general cognitive ability; NDDs, neurodevelopmental disorders (other NDDs includes e.g., communication disorders, specific learning disorders or motor disorders); ToM, theory of mind.

*p < 0.05; **p < 0.01; and ***p < 0.001.

### Between-Pair Associations Between ToM, Other Cognitive Functions and NDDs

Results for linear regressions across pairs with ToM as outcome are shown in Table 3. A higher CC and IQ, but not EF, were associated with a better ToM ability. In addition, female sex and higher age were also associated with a better ToM ability in the three models [ranging between *b* = −4.945 and −5.533 for sex (female sex as reference); and between *b* = 0.493 and 0.687 for age, all *p* < 0.001]. The associations between ToM and other cognitive functions were similar among twin-pairs concordant or discordant for NDDs and TD twin-pairs, see Table 3.

**TABLE 3:** Results from the linear regressions with central coherence, executive function and IQ as predictors of Theory of Mind, measured with the Reading the Mind in the Eyes Test.

		Central coherence^a^	Executive functions^b^	IQ^c^
		b (95% CI)	b (95% CI)	b (95% CI)
All pairs	Between-pair^*d*^	−0.388** (−0.624, −0.151)	0.422 (−0.163, 1.007)	0.265*** (0.166, 0.364)
	Within-pair	−0.282 (−0.592, 0.027)	0.424 (−0.131, 0.980)	0.215* (0.016, 0.413)
	Within-pair MZ	−0.272 (−0.782, 0.239)	0.239 (−0.592, 1.070)	0.281 (−0.121, 0.682)
TD pairs	Between-pair^*d*^	−0.408 (−0.823, 0.007)	0.266 (−0.671, 1.203)	0.331*** (0.218, 0.444)
	Within-pair	−0.382 (−0.834, 0.071)	0.290 (−0.645, 1.227)	0.180 (−0.032, 0.391)
NDD pairs	Between-pair^*d*^	−0.359* (−0.674, −0.043)	0.514 (−0.232, 1.259)	0.224** (0.082, 0.366)
	Within-pair	−0.216 (−0.641, 0.209)	0.496 (−0.194, 1.186)	0.228 (−0.034, 0.490)

^*a*^Measured with the Fragmented Pictures Test. ^*b*^Measured with the Tower Test. ^*c*^Measured with the General ability index from the Wechsler Intelligence Scales for Children or Adults-IV. ^*d*^Between-pair calculations are adjusted for sex and age. NDDs, neurodevelopmental disorders; TD, typically developing.

*p < 0.05; **p < 0.01; and ***p < 0.001.

### Full Model of Between-Pair Associations Between ToM, Other Cognitive Functions and NDDs

When including all cognitive functions (CC, EF, and IQ), diagnoses (ASD, ADHD, and other NDDs), sex and age in the same model as independent variables and ToM as outcome, the association with CC, IQ, female sex and increasing age remained, but the association with a diagnosis of NDD (ASD, ADHD, other NDDs) from the correlation analyses was lost, Table 4.

**TABLE 4:** Results from the linear regressions with central coherence, executive function and IQ as predictors of Theory of Mind as measured with the Reading the Mind in the Eyes Test, also adjusting for diagnoses, sex and age.

	Between-pair	Within-pair
	b (95% CI)	b (95% CI)
Central coherence^a^	−0.250 (−0.485, −0.016)*	−0.188 (−0.459, 0.082)
Executive functions^b^	0.042 (−0.435, 0.518)	0.088 (−0.445, 0.621)
IQ^c^	0.242 (0.149, 0.336)***	0.191 (−0.005, 0.388)
ASD	−2.462 (−6.537, 1.613)	−1.353 (−5.956, 3.250)
ADHD	−2.119 (−5.779, 1.542)	−0.609 (−4.846, 3.628)
Other NDDs	−0.015 (−3.522, 3.493)	0.001 (−3.629, 3.631)
Sex (male)	−4.878 (−7.296, −2.460)***	
Age	0.466 (0.224, 0.707)***	

^*a*^Measured with the Fragmented Pictures Test. ^*b*^Measured with the Tower Test. ^*c*^Measured with the General ability index from the Wechsler Intelligence Scales for Children or Adults-IV. ADHD, attention-deficit/hyperactivity disorder; ASD, autism spectrum disorder; NDDs, Neurodevelopmental disorders (other NDDs includes e.g., communication disorders, specific learning disorders or motor disorders).

*p < 0.05; **p < 0.01; and ***p < 0.001.

### Within-Pair Associations Between ToM and Other Cognitive Functions

As shown in Table 3, the association between CC and ToM was lost within pairs, whereas the association between IQ and ToM remained, although weakened and lost in the MZ subset. When including all cognitive functions (CC, EF, and IQ) and NDD diagnoses (ASD, ADHD, and other NDDs) in the same model, all associations with ToM were lost within the pairs, Table 4.

## Discussion

This is the first study using a co-twin control design to investigate the familial underpinnings between ToM and other cognitive functions in a sample enriched for twins concordant and discordant for NDDs. IQ and CC were associated with ToM ability across pairs. In addition, female sex and higher age were robustly linked with ToM ability in all models. The within-pair analyses attenuated the associations between ToM and other cognitive functions, especially for CC which was then no longer significant, and to some degree also IQ. The results from the MZ within-pair analyses were non-significant, however, these findings were more ambiguous given a broad confidence interval for the estimations, possibly due to a low power. Our results suggest that familial factors shared by the twins, such as genetic background and shared environment, influence the association between CC, IQ, and ToM.

Across the sample, the overarching cognitive functions of CC, EF, and IQ were associated with ToM in the correlation analyses, with small to moderate effect sizes. Comparable associations have been reported in numerous studies. Although no previous research has explored the associations between results on the Reading the Mind in the Eyes Test and the Fragmented Picture test, scores on other tests measuring CC have been associated with Reading the Mind in the Eyes Test (Jarrold et al., 2000), as well as other tests on false belief understanding (Pellicano, 2010), in typically developed and autistic children. Associations between scores on tests assessing EF and ToM have been found in typically developed, autistic and ADHD children (Pellicano, 2010; Devine and Hughes, 2014; Pineda-Alhucema et al., 2018), and a weak association has been reported between the Tower Test and Reading the Mind in the Eyes Test (Ahmed and Miller, 2011; Stubberud, 2017). For IQ, an association has been found with ToM in general (Coyle et al., 2018), and a week association between score on the Wechsler scale and Reading the Mind in the Eyes Test specifically, in both general and NDD populations (Baker et al., 2014). In our study, the correlation with ToM was similar for the Verbal Comprehension and the Perceptual Reasoning index.

According to the developmental approach, these functions or abilities are interrelated and develop in concert with each other (Pellicano, 2010). Moreover, it has been argued that CC and EF precede ToM (Pellicano, 2010; Devine and Hughes, 2014; Mary et al., 2016; Skorich et al., 2016; Wade et al., 2018), which is why we choose to have ToM as an outcome in our study. Accordingly, the association between CC, IQ, and ToM remained in the GEE model when adjusting for sex and age, although the association with EF was lost, indicating a moderating effect of sex and age. Overall sex, and also age, was strongly associated with ToM functions across all models. This finding corroborates previous research using the Reading the Mind in the Eyes Test with females out-performing males (Baron-Cohen et al., 2001, 2015), and is in line with the Empathizing–Systemizing theory, where males are more systemizing and females more empathic, and where autism is understood as showing a more extreme variant of cognition than found on average in males (Baron-Cohen, 2002).

All included NDD diagnoses were correlated with ToM and CC. Especially ASD has been linked to ToM and WCC, where a reduced ability to represent one’s own and others thoughts, emotions and beliefs, has been hypothesized to be an integral part of the social and communication difficulties underlying ASD (Baron-Cohen, 2009; Mazza et al., 2017), whereas a reduced style to integrate stimulus information into a coherent whole has been proposed to underlie autism related talents such as an eye for details (Happé and Frith, 2006). However, in the fully adjusted model across pairs, the associations between specific NDDs and ToM ability were lost. Instead, sex and age, as well as CC and IQ, were the main factors associated with ToM scores. This finding suggests that ToM ability is not uniquely associated with ASD, but rather mediated by other factors that are partly associated with ASD. Furthermore, the associations between ToM and other cognitive functions were similar among twins concordant and discordant for NDDs and TD twin-pairs, indicating that the cognitive functions do not decouple and segregate independently in families with and without NDDs. Similar findings were found by Jarrold et al. (2000) who reported a correlation between WCC and ToM ability in both children with ASD and TD when adjusting for verbal mental age.

Interestingly, neither EF nor IQ were associated with ASD or ADHD in the unadjusted correlations, which is surprising since EF difficulties have been suggested to contribute to the core symptoms of ADHD (Alderson et al., 2007; Kasper et al., 2012), as well as rigid and repetitive behavior in ASD (Demetriou et al., 2019). Furthermore, in a recent review it was concluded that foresighted planning problems, e.g., measured with the Tower Test, were present among children with ASD, as well as among children with comorbid ASD and ADHD (Craig et al., 2016). In our study we used the Tower Test as a proxy for EF, and possibly, the measure of errors of omission and commission may be more sensitive for e.g., ADHD (Craig et al., 2016). In addition, if we would have had a more homogeneous sample of ADHD cases, not obscured by comorbidity and broad age range, the result may have been different.

The associations between CC, IQ, and ToM were attenuated and lost in the within-pair analyses, a finding that demonstrates that familial factors contribute to the associations between these cognitive functions. Familial factors are those shared by family members, i.e., genes and shared environment such as parenting style, maternal conditions during pregnancy and social environment during upbringing. This attenuation was most clear in the association between ToM and CC, reducing the estimate with 27%, followed by a reduction of 19% in the estimates between IQ and ToM. Previous research has also emphasized the heritability for IQ (Plomin and Von Stumm, 2018), as well as for CC in twins with or without eating disorders (Kanakam et al., 2013), whereas heritability estimates for ToM have been modest (Hughes et al., 2005; Ronald et al., 2006). Our finding of an attenuated association within the pairs does not give support for ToM being dependent of CC, as has been suggested previously according to the developmental approach.

The results from the MZ subsample were more ambiguous, with large confidence intervals for the estimates, indicating that the statistical calculations were under-powered. A larger MZ sample would be necessary in order to draw more firm conclusions regarding the influence of familial factors, and differentiating shared environmental from genetic effects would require both larger MZ and DZ samples. Even though, to the best of our knowledge, no previous studies have explicitly explored the association between CC, EF, IQ and ToM using a co-twin control design, there are some studies conducted on siblings. Oerlemans et al. (2013) reported an interrelatedness between SC and EF task performance, but not between SC and CC performance in children with or without ASD. This interrelatedness was found between siblings, i.e., SC in probands was related to EF in their siblings and vice versa, a finding that implies similar familial underpinnings between the SC and EF domain (Oerlemans et al., 2013). This contrasts both our finding on a weak association between ToM and EF, and that shared familial underpinnings are more evident in the ToM and CC association. Our study however, differ in several aspects from Oerlemans et al. (2013), using different statistics, different tests for ToM, EF, and CC, and only the current study included other coexisting NDDs.

The present study has limitations that need to be addressed. First, although this is a reasonably sized study using deeply phenotyped twins (Bölte et al., 2014), our results require replication in even larger samples to ensure sufficient power, especially in the MZ within-pair analyses. Second, since our sample is biased toward MZ twins discordant for NDDs, we did not model quantitative contributions of A (additive genetics), C (common/shared environment), and E (unique environment), which would lead to biased estimates in the twin model fitting. A higher number of discordant pairs, however, increase the sensitivity of the within-pair models. Third, we used a single measure to define the cognitive functions. Other measurements, covering other dimensions of SC, EF, CC, and IQ, may have yielded different findings. Our study relies exclusively on psychological tests, and adding self- or parent rated questionnaires, or more psychological tests, may provide additional information about symptoms and functioning in everyday life. In addition, the Reading the Mind in the Eyes Test has been criticized for measuring emotion recognition rather than ToM ability (Oakley et al., 2016) and being dependent on the participants vocabulary (Olderbak et al., 2015). At the same time, the correlation between verbal abilities and ToM was weak in our sample, and partly adjusted for within the GAI measure. Fourth, comorbidity was common in our sample, with some individuals having more than one NDD. This reduced power in the adjusted model, but also increased the ecological validity since comorbidity is common in the general population as well (Licari et al., 2019). Fifth, our study does not allow inference regarding directionality of the reported associations since it is correlational. Future twin studies should address these gaps by using multiple tasks of cognitive functions and including a larger sample of MZ twins and applying longitudinal designs. Sixth, no correction for multiple testing was made, and if applying Bonferroni correction, based on the number of main analyses, the null hypothesis would have been rejected if *p* < 0.013. However, with this method the likelihood of type II errors is also increased, and it is argued that the best approach is to describe what has been done and why (Perneger, 1998).

To conclude, by using a sample enriched with twins concordant and discordant for NDDs we show that WCC and a lowered general cognitive functioning are associated with a reduced ToM ability, even when NDDs are taken into account. By being the first study utilizing a co-twin control design, we found that the associations between CC, IQ, and ToM were attenuated, demonstrating that familial factors contribute to the association. This finding suggests that shared genetic or environmental factors within the family explain some part of the associations between WCC, general cognitive functioning and ToM. More studies with larger sample sizes are, however, needed to further investigate the specific contributions of genes and environment on the associations. As for the developmental approach, out finding of an attenuated association within the pairs does not give support for ToM being dependent of CC, as has been suggested previously. Rather, both functions may have a common etiological background. Also, given the robust associations between female sex, age, and ToM ability, future studies need to include these factors when assessing ToM. For the future, employing different measures to assess cognitive functions, including ToM, in larger samples of MZ twins would enable us to study the developmental pathways to alterations in ToM in more depth.

## Data Availability Statement

The datasets presented in this article are not readily available because of regulations in the ethical approval and university policies, requiring among others a data sharing agreement. Requests to access the datasets should be directed to SB, sven.bolte@ki.se.

## Ethics Statement

The study involving human participants was reviewed and approved by the Regional Swedish Ethical Review Board in Stockholm and informed consent was obtained from all participants or their caregivers after the nature of the procedure had been fully explained.

## Author Contributions

SB contributed to conception and design of the study. JI and JN performed the statistical analysis. JI wrote the first draft in collaboration with JN. All authors contributed to manuscript revision, read, and approved the submitted version.

## Conflict of Interest

SB in last four years acted as an author, consultant, or lecturer for Medice and Roche, and receives royalties for text books or diagnostic instruments from Huber/Hogrefe, Kohlhammer and UTB. The remaining authors declare that the research was conducted in the absence of any commercial or financial relationships that could be construed as a potential conflict of interest.

## References

[B1] Ahmed F. S. Miller L. S. (2011). Executive function mechanisms of theory of mind. *J. Autism. Dev. Disord*. 4 667–678. 10.1007/s10803-010-1087-7 20811770

[B2] Alderson R. M. Rapport M. D. Kofler M. J. (2007). Attention-deficit/hyperactivity disorder and behavioral inhibition: a meta-analytic review of the stop-signal paradigm. *J. Abnorm. Child. Psychol.* 35 745–758. 10.1007/s10802-007-9131-6 17668315

[B3] Anckarsäter H. Lundstrom S. Kollberg L. Kerekes N. Palm C. Carlstrom E. (2011). The Child and Adolescent Twin Study in Sweden (CATSS). *Twin. Res. Hum. Genet.* 14 495–508. 10.1375/twin.14.6.495 22506305

[B4] Atherton G. Cross L. (2018). Seeing More Than Human: Autism and Anthropomorphic Theory of Mind. *Front. Psychol.* 9:528. 10.3389/fpsyg.2018.00528 29755383 PMC5932358

[B5] Baker C. A. Peterson E. Pulos S. Kirkland R. A. (2014). Eyes and IQ: A meta-analysis of the relationship between intelligence and “Reading the Mind in the Eyes”. *Intelligence* 44 78–92. 10.1016/j.intell.2014.03.001

[B6] Baribeau D. A. Doyle-Thomas K. A. Dupuis A. Iaboni A. Crosbie J. Mcginn H. (2015). Examining and comparing social perception abilities across childhood-onset neurodevelopmental disorders. *J. Am. Acad. Child. Adolesc. Psychiatry.* 54:e471. 10.1016/j.jaac.2015.03.016 26004663

[B7] Baron-Cohen S. (2002). The extreme male brain theory of autism. *Trends. Cogn. Sci.* 6 248–254. 10.1016/s1364-6613(02)01904-6 12039606

[B8] Baron-Cohen S. (2009). Autism: the empathizing-systemizing (E-S) theory. *Ann. N. Y. Acad. Sci.* 1156 68–80. 10.1111/j.1749-6632.2009.04467.x 19338503

[B9] Baron-Cohen S. Bowen D. C. Holt R. J. Allison C. Auyeung B. Lombardo M. V. (2015). The “Reading the Mind in the Eyes” Test: Complete Absence of Typical Sex Difference in ~400 Men and Women with Autism. *PLoS. One.* 10:e0136521. 10.1371/journal.pone.0136521 26313946 PMC4552377

[B10] Baron-Cohen S. Jolliffe T. Mortimore C. Robertson M. (1997). Another advanced test of theory of mind: evidence from very high functioning adults with autism or Asperger syndrome. *J. Child. Psychol. Psychiatry* 38, 813–822. 10.1111/j.1469-7610.1997.tb01599.x 9363580

[B11] Baron-Cohen S. Wheelwright S. Hill J. Raste Y. Plumb I. (2001). The “Reading the Mind in the Eyes”. *J. Child. Psychol. Psychiatry.* 42 241–251. 11280420

[B12] Bölte S. Ciaramidaro A. Schlitt S. Hainz D. Kliemann D. Beyer A. (2015). Training-induced plasticity of the social brain in autism spectrum disorder. *Br. J. Psychiatry.* 207 149–157. 10.1192/bjp.bp.113.143784 25792694

[B13] Bölte S. Willfors C. Berggren S. Norberg J. Poltrago L. Mevel K. (2014). The Roots of Autism and ADHD Twin Study in Sweden (RATSS). *Twin. Res. Hum. Genet.* 17 164–176. 10.1017/thg.2014.12 24735654

[B14] Booth R. D. L. Happé F. G. E. (2018). Evidence of Reduced Global Processing in Autism Spectrum Disorder. *J. Autism. Dev. Disord.* 48 1397–1408. 10.1007/s10803-016-2724-6 26864159 PMC5861162

[B15] Brunsdon V. E. Colvert E. Ames C. Garnett T. Gillan N. Hallett V. (2015). Exploring the cognitive features in children with autism spectrum disorder, their co-twins, and typically developing children within a population-based sample. *J. Child. Psychol. Psychiatry.* 56 893–902. 10.1111/jcpp.12362 25418509

[B16] Brunsdon V. E. Happé F. (2014). Exploring the ‘fractionation’ of autism at the cognitive level. *Autism* 18 17–30. 10.1177/1362361313499456 24126870

[B17] Callenmark B. Kjellin L. Ronnqvist L. Bölte S. (2014). Explicit versus implicit social cognition testing in autism spectrum disorder. *Autism* 18 684–693. 10.1177/1362361313492393 24104519 PMC4230543

[B18] Conners C. K. (2008). *Conners 3rd Edition Manual.* Toronto: Multi-Health Systems.

[B19] Constantino J. N. Gruber C. P. (2005). *Social Responsiveness Scale (SRS).* Los Angeles, CA: Western Psychological Services.

[B20] Coyle T. R. Elpers K. E. Gonzalez M. C. Freeman J. Baggio J. A. (2018). General Intelligence (g), ACT Scores, and Theory of Mind: (ACT)g Predicts Limited Variance Among Theory of Mind Tests. *Intelligence* 71 85–91. 10.1016/j.intell.2018.10.006

[B21] Craig F. Margari F. Legrottaglie A. R. Palumbi R. De Giambattista C. Margari L. (2016). A review of executive function deficits in autism spectrum disorder and attention-deficit/hyperactivity disorder. *Neuropsychiatr. Dis. Treat.* 12 1191–1202. 10.2147/NDT.S104620 27274255 PMC4869784

[B22] Delis D. C. Kaplan E. Kramer J. H. (2001). *The Delis-Kaplan Executive Function System: Examiner’s Manual*. San Antonio, TX: The Psychological Corporation.

[B23] Demetriou E. A. Demayo M. M. Guastella A. J. (2019). Executive Function in Autism Spectrum Disorder: History, Theoretical Models, Empirical Findings, and Potential as an Endophenotype. *Front. Psychiatry.* 10:753. 10.3389/fpsyt.2019.00753 31780959 PMC6859507

[B24] Devine R. T. Hughes C. (2014). Relations between false belief understanding and executive function in early childhood: a meta-analysis. *Child. Dev.* 85 1777–1794. 10.1111/cdev.12237 24605760

[B25] Diamond A. (2013). Executive functions. *Annu. Rev. Psychol*. 64 135–168. 10.1146/annurev-psych-113011-143750 23020641 PMC4084861

[B26] Grove R. Baillie A. Allison C. Baron-Cohen S. Hoekstra R. A. (2014). The latent structure of cognitive and emotional empathy in individuals with autism, first-degree relatives and typical individuals. *Mol. Autism.* 5:42. 10.1186/2040-2392-5-42 25101164 PMC4123248

[B27] Hallerbäck M. U. Lugnegård T. Hjärthag F. Gillberg C. (2009). The reading the mind in the eyes test: test-retest reliability of a Swedish version. *Cogn. Neuropsychiatry* 14, 127–143. 10.1080/13546800902901518 19370436

[B28] Hannelius U. Gherman L. Makela V. V. Lindstedt A. Zucchelli M. Lagerberg C. (2007). Large-scale zygosity testing using single nucleotide polymorphisms. *Twin. Res. Hum. Genet.* 10 604–625. 10.1375/twin.10.4.604 17708702

[B29] Happé F. Cook J. L. Bird G. (2017). The Structure of Social Cognition: In(ter)dependence of Sociocognitive Processes. *Annu. Rev. Psychol.* 68 243–267. 10.1146/annurev-psych-010416-044046 27687121

[B30] Happé F. Frith U. (2006). The weak coherence account: detail-focused cognitive style in autism spectrum disorders. *J. Autism. Dev. Disord.* 36 5–25. 10.1007/s10803-005-0039-0 16450045

[B31] Happé F. Ronald A. (2008). The ‘fractionable autism triad’: a review of evidence from behavioural, genetic, cognitive and neural research. *Neuropsychol. Rev.* 18 287–304. 10.1007/s11065-008-9076-8 18956240

[B32] Hughes C. Jaffee S. R. Happe F. Taylor A. Caspi A. Moffitt T. E. (2005). Origins of individual differences in theory of mind: from nature to nurture? *Child. Dev.* 76 356–370. 10.1111/j.1467-8624.2005.00850.x 15784087

[B33] Isaksson J. Taylor M. J. Lundin K. Neufeld J. Bölte S. (2019a). Familial confounding on the ability to read minds: A co-twin control study. *Autism* 23 1948–1956. 10.1177/1362361319836380 30895802

[B34] Isaksson J. Van’t Westeinde A. Cauvet E. Kuja-Halkola R. Lundin K. Neufeld J. (2019b). Social Cognition in Autism and Other Neurodevelopmental Disorders: A Co-twin Control Study. *J. Autism. Dev. Disord.* 49 2838–2848. 10.1007/s10803-019-04001-4 30972652 PMC6606667

[B35] Jarrold C. Butler D. W. Cottingham E. M. Jimenez F. (2000). Linking theory of mind and central coherence in autism and in the general population. *Dev. Psychol*. 36 126–138. 10.1037//0012-1649.36.1.126 10645750

[B36] Kanakam N. Raoult C. Collier D. Treasure J. (2013). Set shifting and central coherence as neurocognitive endophenotypes in eating disorders: a preliminary investigation in twins. *World. J. Biol. Psychiatry.* 14 464–475. 10.3109/15622975.2012.665478 22630167

[B37] Kasper L. J. Alderson R. M. Hudec K. L. (2012). Moderators of working memory deficits in children with attention-deficit/hyperactivity disorder (ADHD): a meta-analytic review. *Clin. Psychol. Rev.* 32 605–617. 10.1016/j.cpr.2012.07.001 22917740

[B38] Kaufman J. Birmaher B. Brent D. Rao U. Flynn C. Moreci P. (1997). Schedule for Affective Disorders and Schizophrenia for School-Age Children-Present and Lifetime Version (K-SADS-PL): initial reliability and validity data. *J. Am. Acad. Child. Adolesc. Psychiatry.* 36 980–988. 10.1097/00004583-199707000-00021 9204677

[B39] Kessler J. Schaaf A. Mielke R. (1993). *Der Fragmentierte Bildertest (FBT).* G€ottingen, Germany: Hogrefe.

[B40] Kooij J. J. S. Francken M. H. (2010). “Diagnostic interview for ADHD in adults 2.0 (DIVA 2.0),” in *Adult ADHD: Diagnostic assessment and treatment*, ed. Kooij J. J. S. (Amsterdam: Pearson Assessment), 2–19.

[B41] Licari M. K. Finlay-Jones A. Reynolds J. E. Alvares G. A. Spittle A. J. Downs J. (2019). The brain basis of comorbidity in neurodevelopmental disorders. *Curr. Dev. Disord. Rep* 6 9–18. 10.1007/s40474-019-0156-7

[B42] Lord C. Rutter M. DiLavore P. Risi S. Gotham K. Bishop S. (2012). *Autism diagnostic observation schedule–2nd edition (ADOS-2).* Los Angeles, CA: Western Psychological Corporation.

[B43] Losh M. Adolphs R. Poe M. D. Couture S. Penn D. Baranek G. T. (2009). Neuropsychological profile of autism and the broad autism phenotype. *Arch. Gen. Psychiatry.* 66 518–526. 10.1001/archgenpsychiatry.2009.34 19414711 PMC2699548

[B44] Mary A. Slama H. Mousty P. Massat I. Capiau T. Drabs V. (2016). Executive and attentional contributions to Theory of Mind deficit in attention deficit/hyperactivity disorder (ADHD). *Child. Neuropsychol.* 22 345–365. 10.1080/09297049.2015.1012491 25763856

[B45] Matson J. L. Shoemaker M. (2009). Intellectual disability and its relationship to autism spectrum disorders. *Res. Dev. Disabil.* 30 1107–1114. 10.1016/j.ridd.2009.06.003 19604668

[B46] Mazza M. Mariano M. Peretti S. Masedu F. Pino M. C. Valenti M. (2017). The Role of Theory of Mind on Social Information Processing in Children With Autism Spectrum Disorders: A Mediation Analysis. *J. Autism. Dev. Disord.* 47 1369–1379. 10.1007/s10803-017-3069-5 28213839

[B47] Morgan B. Maybery M. Durkin K. (2003). Weak central coherence, poor joint attention, and low verbal ability: independent deficits in early autism. *Devel. Psychol.* 39, 646–656. 10.1037/0012-1649.39.4.646 12859119

[B48] Neufeld J. Hagström A. Van’t Westeinde A. Lundin K. Cauvet É Willfors C. (2020). Global and local visual processing in autism–a co−twin−control study. *J. Child Psychol. Psychiatry* 61 470–479. 10.1111/jcpp.13120 31452200 PMC7155117

[B49] Neuhaus J. M. McCulloch E. (2006). Separating betweenand within-cluster covariate effects by using conditional and partitioning methods. *Stat. Methodol.* 68 859–872. 10.1111/j.1467-9868.2006.00570.x

[B50] Oakley B. F. M. Brewer R. Bird G. Catmur C. (2016). Theory of mind is not theory of emotion: A cautionary note on the Reading the Mind in the Eyes Test. *J. Abnorm. Psychol.* 125 818–823. 10.1037/abn0000182 27505409 PMC4976760

[B51] Oerlemans A. M. Droste K. Van Steijn D. J. De Sonneville L. M. Buitelaar J. K. Rommelse N. N. (2013). Co-segregation of social cognition, executive function and local processing style in children with ASD, their siblings and normal controls. *J. Autism. Dev. Disord.* 43 2764–2778. 10.1007/s10803-013-1807-x 23532348

[B52] Olderbak S. Wilhelm O. Olaru G. Geiger M. Brenneman M. W. Roberts R. D. (2015). A psychometric analysis of the reading the mind in the eyes test: toward a brief form for research and applied settings. *Front. Psychol.* 6:1503. 10.3389/fpsyg.2015.01503 26500578 PMC4593947

[B53] Patros C. H. G. Tarle S. J. Alderson R. M. Lea S. E. Arrington E. F. (2019). Planning deficits in children with attention-deficit/hyperactivity disorder (ADHD): A meta-analytic review of tower task performance. *Neuropsychology* 33 425–444. 10.1037/neu0000531 30688493

[B54] Pellicano E. (2010). Individual differences in executive function and central coherence predict developmental changes in theory of mind in autism. *Dev. Psychol.* 46 530–544. 10.1037/a0018287 20210511

[B55] Pellicano E. Maybery M. Durkin K. Maley A. (2006). Multiple cognitive capabilities/deficits in children with an autism spectrum disorder: “weak” central coherence and its relationship to theory of mind and executive control. *Dev. Psychopathol.* 18 77–98. 10.1017/S0954579406060056 16478553

[B56] Perneger T. V. (1998). What’s wrong with Bonferroni adjustments. *BMJ* 316 1236–1238. 10.1136/bmj.316.7139.1236 9553006 PMC1112991

[B57] Pineda-Alhucema W. Aristizabal E. Escudero-Cabarcas J. Acosta-Lopez J. E. Velez J. I. (2018). Executive Function and Theory of Mind in Children with ADHD: a Systematic Review. *Neuropsychol. Rev.* 28 341–358. 10.1007/s11065-018-9381-9 30168020

[B58] Plomin R. Von Stumm S. (2018). The new genetics of intelligence. *Nat. Rev. Genet.* 19 148–159. 10.1038/nrg.2017.104 29335645 PMC5985927

[B59] Razza R. A. (2009). Associations among False-belief Understanding. Executive Function, and Social Competence: A Longitudinal Analysis. *J. Appl. Dev. Psychol*. 30 332–343. 10.1016/j.appdev.2008.12.020 20161159 PMC2735755

[B60] Ronald A. Viding E. Happe F. Plomin R. (2006). Individual differences in theory of mind ability in middle childhood and links with verbal ability and autistic traits: a twin study. *Soc. Neurosci.* 1 412–425. 10.1080/17470910601068088 18633802

[B61] Rutter M. Le Couteur A. Lord C. (2003). *ADI-R: Autism Diagnostic Interview Revised.* Los Angeles, CA: Western Psychological Services.

[B62] Sachse M. Schlitt S. Hainz D. Ciaramidaro A. Walter H. Poustka F. (2014). Facial emotion recognition in paranoid schizophrenia and autism spectrum disorder. *Schizophr. Res.* 159 509–514. 10.1016/j.schres.2014.08.030 25278104

[B63] Saklofske D. H. Zhu J. Coalson D. L. Raiford S. E. Weiss L. G. (2010). Cognitive proficiency index for the Canadian edition of the Wechsler Intelligence Scale for Children—Fourth Edition. *Can. J. Sch. Psychol.* 25 277–286. 10.1177/0829573510380539

[B64] Scheurich A. Fellgiebel A. Muller M. J. Poustka F. Bölte S. (2010). [Does the Fragmented Images Test measure locally oriented visual processing in autism spectrum disorders?] Z. Kinder. Jugendpsychiatr. *Psychother* 38 103–110. 10.1024/1422-4917.a000017 20200827

[B65] Shakoor S. Jaffee S. R. Bowes L. Ouellet-Morin I. Andreou P. Happe F. (2012). A prospective longitudinal study of children’s theory of mind and adolescent involvement in bullying. *J. Child. Psychol. Psychiatry.* 53 254–261. 10.1111/j.1469-7610.2011.02488.x 22081896 PMC3272094

[B66] Skorich D. P. May A. R. Talipski L. A. Hall M. H. Dolstra A. J. Gash T. B. (2016). Is Social Categorization the Missing Link Between Weak Central Coherence and Mental State Inference Abilities in Autism? Preliminary Evidence from a General Population Sample. *J. Autism. Dev. Disord.* 46 862–881. 10.1007/s10803-015-2623-2 26438641

[B67] Slaughter V. Imuta K. Peterson C. C. Henry J. D. (2015). Meta-Analysis of Theory of Mind and Peer Popularity in the Preschool and Early School Years. *Child. Dev.* 86 1159–1174. 10.1111/cdev.12372 25874384

[B68] Smit L. Knoors H. Hermans D. Verhoeven L. Vissers C. (2019). The Interplay Between Theory of Mind and Social Emotional Functioning in Adolescents With Communication and Language Problems. *Front. Psychol.* 10:1488. 10.3389/fpsyg.2019.01488 31333537 PMC6616194

[B69] Söderstrand P. (2006). *Eyes test Swedish version. Neuropsychiatric Unit, Psychiatric Clinic, Hospital of Bora’s.* Available online at: https://www.autismresearchcentre.com/arc_tests (accessed May 12, 2020).

[B70] Stubberud J. (2017). Theory of mind in spina bifida: relationship with intellectual and executive functioning. *Scand. J. Psychol.* 58 379–388. 10.1111/sjop.12390 28901576

[B71] Vellante M. Baron-Cohen S. Melis M. Marrone M. Petretto D. R. Masala C. (2013). The “Reading the Mind in the Eyes” test: Systematic review of psychometric properties and a validation study in Italy. *Cogn. Neuropsychiatry*. 18 326–354. 10.1080/13546805.2012.721728 23106125 PMC6345369

[B72] Wade M. Prime H. Jenkins J. M. Yeates K. O. Williams T. Lee K. (2018). On the relation between theory of mind and executive functioning: A developmental cognitive neuroscience perspective. *Psychon. Bull. Rev.* 25 2119–2140. 10.3758/s13423-018-1459-0 29619648

[B73] Zander E. Karlsson C. Bölte S. (2011). *Child Eyes Test—Svensk. Karolinska Institutet Center of Neurodevelopmental Disorders.* Available online at: https://www.autismresearchcentre.com/arc_tests (accessed May 12, 2020).

