# Supplementary material for: What’s the Link Between Theory of Mind and Other Cognitive Abilities – A Co-twin Control Design of Neurodevelopmental Disorders
Source: Front Psychol. 2021 Jun 8;12:575100. doi: 10.3389/fpsyg.2021.575100 (PMC8217460; doi:10.3389/fpsyg.2021.575100)
Supplement: Supplementary file 1 [file Table_1.docx]

**Supplementary Table 1** Correlations between Theory of Mind (ToM), central coherence (CC) and subscales on the Wechsler Intelligence Scales for Children or Adults-IV for General ability index.

|  | ToM | CC | EF |
| --- | --- | --- | --- |
| VCI | .295*** | -.179** | .166** |
| Comprehension/Information | .280*** | -.209*** | .121* |
| Vocabulary | .280*** | -.147** | .106 |
| Similarities | .234*** | -.162** | .215*** |
| PRI | .200*** | -.134* | .279*** |
| Block Design | .099 | -.046*** | .245*** |
| Matrix Reasoning | .255*** | -.237*** | .233*** |
| Picture Concepts | .154** | -.217*** | .133** |

Note: Correlations between continuous variables were calculated using Spearman’s Correlation Coefficient.

* p < .05; ** p < .01; *** p < .001
